# Signaling pathway mechanisms in pancreatic ductal adenocarcinoma tumor microenvironment and emerging targeting strategies for improved prognosis

**DOI:** 10.3389/or.2026.1814540

**Published:** 2026-05-29

**Authors:** Tosin Akinsipe, Caroline O. Odewumi, Lekan Latinwo, Omorose O. Aghimien, Musiliyu A. Musa, Philip O. Igbinoba, Anthony L. Simons, Monica O. Aghimien

**Affiliations:** 1 Department of Biological Sciences, University of Pittsburgh, Pittsburgh, PA, United States; 2 Department of Biological Sciences, Florida A&M University, Tallahassee, FL, United States; 3 Department of Chemistry, Florida A&M University, Tallahassee, FL, United States; 4 Department of Health Sciences, Florida A&M University, Tallahassee, FL, United States

**Keywords:** improved prognosis, pancreatic cancer, PDAC, polymeric nanoparticles, signaling pathway, targeted delivery, tumor microenvironment

## Abstract

Pancreatic ductal adenocarcinoma (PDAC) remains one of the most lethal malignancies, characterized by a dense desmoplastic stroma and a profoundly immunosuppressive tumor microenvironment (TME). The TME plays a major role in tumor progression, metastasis, and resistance to conventional therapies through a network of dysregulated signaling pathways, including KRAS, PI3K/AKT/mTOR, Raf/MAPK/ERK, TGF-β, NF-κB, Notch and Hedgehog. Moreover, cellular components such as cancer-associated fibroblasts (CAFs), tumor-associated macrophages (TAMs), and regulatory T cells (Tregs) drive immune evasion and therapeutic resistance via cytokine signaling axes, including IL-6/STAT3 and CXCL12/CXCR4. Recent advances in nanomedicine have introduced polymeric nanoparticles as promising delivery vehicles for targeted disruption of these aberrant pathways. Polymeric nanoparticles are engineered to enhance bioavailability, tissue penetration, and selective delivery, co-deliver small-molecule inhibitors, siRNA, or immunomodulatory agents directly to the TME. This approach offers a strategy to overcome biological barriers, reprogram the stroma, and sensitize tumors to immunotherapy and chemotherapy. This review comprehensively examines the signaling mechanisms of PDAC in the TME, discusses current therapeutic strategies targeting these pathways, highlights challenges, including resistance and adverse effects, and explores future directions to optimize pancreatic cancer treatment by modulating this key signaling axis with nanomedicine.

## Introduction

Pancreatic cancer is a fatal and clinically challenging cancer that is estimated to cause about 51,980 deaths among 67,440 diagnoses in 2025 (1). This makes pancreatic cancer one of the leading causes of cancer deaths. The two main pancreatic cancer types are pancreatic cancer adenocarcinoma (PDAC), and pancreatic neuroendocrine tumors (PNETs), with PDAC making up about 90%–95% of pancreatic cancer and PNETs about 5%–10% (2, 3). Early-stage pancreatic cancer may not exhibit any symptoms, making detection challenging. Effective treatment of pancreatic cancer is difficult since it is frequently discovered at an advanced stage. The aggressiveness of the disease and its late-stage detection contribute to a poor prognosis, with an overall 5-year survival rate (4, 5). The complexity of pancreatic cancer’s biology, as well as its aggressiveness, makes treatment difficult. For instance, PDAC is characterized by its extensive desmoplastic stroma, which creates a fibrotic environment that impedes effective drug delivery and immune cell infiltration (6, 7). Furthermore, dysregulated signaling pathways, notably KRAS (PI3K/AKT/mTOR, Raf/MEK/ERK), Notch, TGF-β, Hedgehog, etc., drive PDAC progression, complicating treatment. Detection of pancreatic cancer in the advanced stage limits conventional treatment methods. Pancreatic cancer is mostly detected when it is unresectable, because tumors have locally advanced or metastasized (8).

Despite these challenges, significant advances have been made in understanding the mechanisms underlying PDAC. Research into the TME, and its impact on pathways that shape PDAC’s progression, immune landscape and metastasis has unveiled new opportunities for therapeutic interventions (9). In recent times, a significant portion of success has been attributed to the exceptional advantages that polymer-based nanoparticles offer, such as drug delivery, drug efflux bypass, protection against degradation, enhanced pharmacokinetics, and controlled release, among others (10, 11). Ongoing efforts focus on modulating the TME by developing scope for innovative precision therapies using polymer nanoparticles for clinical translation (12, 13).

This review provides a comprehensive overview of PDAC signaling pathways, with an emphasis on their influence on the TME. By examining recent advancements and ongoing research, we aim to offer insights into the signaling mechanisms in the PDAC’s TME that contribute to the tumor progression and treatment-resistant characteristics and how they can be effectively tackled by leveraging the unique potential of polymer-based nanoparticles for improved patient outcomes.

## Kras pathway

**FIGURE 1:**
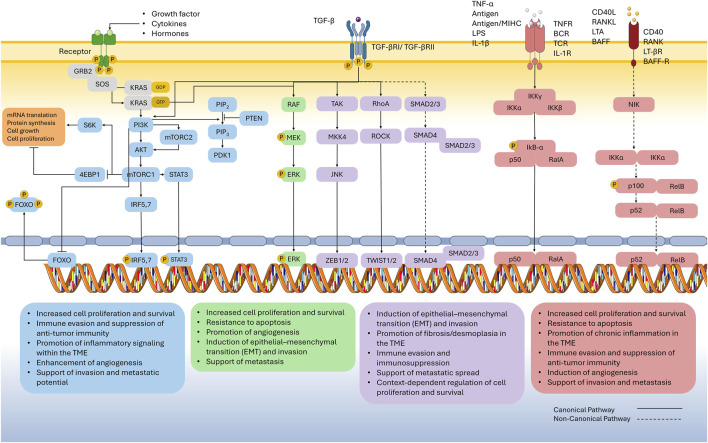
Major signaling pathways in PDAC TME. Major signaling cascades involved in PDAC progression, survival, and therapeutic resistance. Mutant KRAS, a hallmark of PDAC, acts as an upstream driver that activates multiple downstream pathways. The PI3K/AKT/mTOR pathway promotes cell survival, metabolism, and proliferation. The RAF/MAPK/ERK pathway regulates gene expression, cell cycle progression, and differentiation. Additionally, the NF-κB pathway, often activated by inflammatory cytokines and oncogenic stress, maintaining chronic inflammation, immune evasion, and tumor growth. Dysregulation of these pathways enhances tumor aggressiveness and presents challenges for targeted therapy.

KRAS family members encode small GTP-binding proteins in the cytoplasm that help regulate cell cycle progression. A KRAS mutation is among the earliest genetic changes in pancreatic cancer development. The KRAS oncogene, located on chromosome 12p, is activated by a point mutation at codon 2 in over 90% of pancreatic cancers (14). KRAS mutations drive cancer cell proliferation, metabolic reprogramming, immune bypass, and therapy resistance in PDAC, thereby playing a key role in the progression of the disease. KRAS regulates multiple downstream effectors to remodel the PDAC TME (15, 16). Downstream pathways, PI3K/AKT/mTOR and Raf/MAPK/ERK (Figure 1), are the two most prominent pathways induced by KRAS to trigger acinar–to–ductal metaplasia and promote an immunosuppressive TME (17). In the TME, KRAS signaling through the PI3K/AKT/mTOR and Raf/MAPK/ERK pathways contributes to immune tolerance by recruiting myeloid-derived suppressor cells (MDSCs) and Tregs (18, 19). Likewise, KRAS stimulates the release of proinflammatory growth factors and cytokines that attract CAFs and immune cells to the stroma, thereby contributing to fibrotic stroma formation and ECM remodeling (20, 21). Studies have shown that KRAS signaling in PDAC increases the secretion of the pro-inflammatory cytokine interleukin-6 (IL-6) (20, 22, 23). IL-6 drives tumorigenesis by activating Janus-activated kinase 1 (JAK1) and triggering the phosphorylation of signal transducer and activator of transcription 3 (STAT3) (20, 24). In pancreatic acinar cells, KRAS induces M1 macrophages to produce stroma-degrading enzymes, such as matrix metalloproteinase-9, and cytokines, such as tumor necrosis factor (TNF), to facilitate an inflammatory TME (25, 26). Other inflammatory cytokines include IL-1α/β, IL4R, IL-8, IL-10, IL-17, CXCR1, CXCR2 (23, 25, 27–29). Given the importance of the KRAS mutation in pancreatic tumor progression, most therapeutic studies have focused on targeting it. Below are highlighted two major downstream pathways in KRAS signaling: P13/AKT/mTOR and Raf/MAPK/ERK.

### PI3K/AKT/mTOR signaling pathway

The PI3K family consists of Class I (subdivided into IA and IB), II, and III isoforms, with Class IA members p110α, p110β, and p110δ being the most frequently associated with cancer (30). Meanwhile, mTOR is a major integrator of signals related to nutrient status, energy, and growth factors. It operates through two unique complexes, mTORC1 and mTORC2. These complexes regulate critical processes such as protein synthesis, autophagy suppression, and cellular metabolism (31, 32). The PI3K/AKT/mTOR pathway can be initiated by receptor tyrosine kinases (RTKs), G-protein coupled receptors, or oncogenic mutations. These drivers convert phosphatidylinositol 4,5-bisphosphate (PIP2) into phosphatidylinositol 3,4,5-trisphosphate (PIP3), which facilitates the recruitment of AKT to the plasma membrane (Figure 1). There, AKT is activated by PDK1 and mTORC2. Once activated, AKT modulates a broad range of downstream targets, including mTOR1, a crucial effector in cell cycle progression, apoptosis suppression, and metabolic control (33). Due to its major role in maintaining cellular and systemic homeostasis, dysregulation of this pathway is a major contributor to oncogenesis. Dysregulation of PI.

3K/AKT/mTOR promotes metabolic reprogramming, which is essential for cancer cell proliferation and resistance to apoptosis (19, 34). Its abnormal activation is frequently observed across various cancers, typically as a result of mutations in PIK3CA, overexpression of AKT or loss of PTEN (35). Also, stimulation by upstream receptors, including EGFR, and cytokine receptors, is a probable cause (30). The PI3K/AKT/mTOR signaling pathway promotes PDAC tumor cell proliferation and survival by triggering downstream effectors that suppress apoptosis and stimulate protein synthesis. This drives more aggressive tumor growth, which, in turn, continually reshapes the TME (36). In PDAC, targeting PI3K/AKT/mTOR pathway may reduce cancer proliferation, migration and survival (37). Additionally, targeting this pathway could improve the effectiveness of tumor immunosurveillance (38). Inhibitors such as PI3K inhibitors (e.g., pictilisib, buparlisib) (39, 40), AKT inhibitors (e.g., ipatasertib) (41) and mTOR inhibitors (e.g., everolimus, temsirolimus) (42–44) have been developed. Despite the advancement of several inhibitors of the PI3K–AKT–mTOR signaling pathway through various stages of clinical translation, only everolimus (45–47), an allosteric inhibitor of mTORC1/2, and temsirolimus (48, 49), an FKBP-12 inhibitor have been approved for use (50). Moreover, off-target effects, increased toxicity, and reduced effectiveness of a single drug are barriers to their use (51). This calls for exploring more effective approaches, such as using nanoparticles for targeted drug delivery and controlled release to sustain efficacy.

Targeting the PI3K/AKT/mTOR signaling pathway with polymer-based nanoparticles is currently under active investigation for clinical translation. For instance, PLGA-PEG nanoparticles loaded with PI3K inhibitors showed enhanced tumor accumulation and reduced tumor growth in PDAC mouse models (52, 53). In a study, structures of PI3K inhibitors were modified with a cholesterol-based derivative to enable the formation of supramolecular nanoassembly with L-α-phosphatidylcholine and DSPE-PEG. Their anti-tumor efficacy was assessed *in vivo* using ovarian cancer and 4T1 breast cancer models. *In vivo* studies demonstrated that these nanoparticles induced sustained inhibition of mTOR, Akt, S6K, and 4EBP phosphorylation. This prolonged signaling suppression correlated with enhanced antitumor activity and improved survival compared with conventional PI3K inhibitors, such as PI103 and PI828 (54). In another study, the encapsulation of quercetin within mesoporous SBA-15 nanoparticles (QUE-SBA-15 NPs) against NSCLS increased caspase-9 expression, suggesting enhanced apoptosis via the PI3K/AKT/mTOR signaling pathway, compared to unencapsulated quercetin (55). In cancer, overactivation of the PI3K/AKT/mTOR pathway suppresses autophagy, thereby promoting tumor development. Moreover, in some cases, autophagy can support cancer cell survival under therapy-induced stress. A silymarin-based selenium nanoparticle (Si-SeNP) was synthesized and initially evaluated across four different cancer cell lines. The findings indicated that Si-SeNPs activated autophagic flux in the human gastric adenocarcinoma cell line by inhibiting the PI3K/AKT/mTOR pathway, thereby facilitating apoptosis. Furthermore, Nimbolide, a phytochemical that targets pancreatic cancer stem cells (PSCs), was encapsulated in PLGA. When Nimbolide was molecularly docked in the PI3K/AKT/mTOR pathway, it formed a high binding affinity with AKT and mTOR. The *in vitro* results revealed that PLGA encapsulated Nimbolide can induce a robust mesenchymal-to-epithelial transition of pancreatic cancer cell aggregates resulting in the loss of self-renewal properties and multidrug resistance of the cancer cell. This study demonstrates that encapsulating Nimbolide in PLGA nanoparticles increases its therapeutic efficacy against pancreatic cancer cell aggregates. Thereby rendering Nimbolide in nanoparticles more active than unencapsulated Nimbolide (56). The use of nanoparticles to deliver PI3Kγ inhibitor can also help reprogram immune suppression in the TME. When IPI-549, a PI3K inhibitor, was loaded in a nanoparticle and injected into a tumor-bearing mouse, there was significant tumor reduction. Encapsulated IPI-549 significantly reduced both the number and percentage of immunosuppressive cells, MDSCs, and Breg cells compared with unencapsulated IPI-549 (57). Also, PDAC’s acidic TME pH, which supports drug resistance, can be targeted by incorporating chitosan nanoparticles (58).

In other experiments targeting PI3K/AKT/mTOR, Gonzalez-Valdivieso et al. developed self-assembling Elastin-Like Recombinamer (ELR) nanoparticles capable of delivering Akt peptide inhibitors. In both PDAC cell lines and patient-derived xenograft models, these nanoparticles localized to lysosomes, inhibited Akt phosphorylation, disrupted downstream NF-κB signaling, and activated caspase-3–mediated apoptosis (59, 60). The platform also demonstrated extended circulation time and minimal *in vivo* toxicity, underscoring its promise as a targeted nanocarrier with optimized ADME properties within the PDAC TME. Armando Lucero-Acuña et al. encapsulated the allosteric Akt/PDK1 inhibitor PH-427 in PLGA nanoparticles. In an orthotopic MiaPaCa-2 model bearing mutant KRAS, PH-427-loaded nanoparticles achieved sustained drug release for over 30 days, enhanced cellular uptake, and significantly reduced tumor burden compared to the free drug (61). Several other studies have explored co-delivery strategies involving mTOR inhibitors and chemotherapeutics. Although not limited to PDAC, these studies provide important insights into how combination therapies can work more effectively together. For example, nanocarriers that co-deliver rapamycin (an mTOR inhibitor) and paclitaxel have been shown to maintain optimal drug ratios, improve tumor targeting, and enhance cancer cell death by blocking Akt feedback activation (62). Combinatorial therapy, such as loading an mTOR inhibitor and a PD-L1 antibody in a nanoparticle, can be explored to modulate PDAC’s immune microenvironment (63). PLGA-PEG micelles co-loaded with paclitaxel and everolimus achieved dual inhibition of the Warburg effect by targeting the PI3K/Akt/mTOR pathway, thereby improving tumor targeting and therapeutic efficacy (64, 65).

Future research should aim to refine dual-pathway nanocarrier systems to deliver one or more drugs targeting multiple pathways, thereby improving targeting specificity while reducing side effects. It will also be crucial to carry out thorough *in vivo* toxicity studies to pave the way for clinical application. Preclinical studies have already shown promising results, with dual PI3K/mTOR targeting demonstrating effectiveness in orthotopic PDAC models. For example, the dual inhibitor NVP-BEZ235 effectively suppressed Akt activity and downstream p70S6K phosphorylation, reinforcing the potential of combination strategies to overcome compensatory feedback loops (66). However, systemic toxicity remains a major hurdle in clinical application, emphasizing the need for nanoparticle-mediated delivery systems that can localize treatment and minimize off-target effects.

### Raf/MAPK/ERK signaling pathway

The Raf/MAPK/ERK pathway, commonly known as the MAPK/ERK pathway, is a highly conserved signaling cascade (Figure 1). It conveys extracellular signals from Receptor tyrosine kinases (RTKs) to the nucleus, to regulate gene expression process critical for determining cell fate (67). Mutations in components such as RAS, RAF (particularly BRAF), or upstream receptors can disrupt this pathway. This can lead to the development of several cancers including PDAC (68). The Raf/MAPK/ERK pathway is initiated when growth factors bind to RTKs such as EGFR, KRAS, and FGFR, leading to receptor dimerization and autophosphorylation. Resultant binding sites for adaptor proteins such as Grb2 and guanine nucleotide exchange factors (GEFs) are created, facilitating the exchange of GDP for GTP on RAS and, in turn, activating it. Once activated, RAS recruits and stimulates Raf kinases (ARAF, BRAF, CRAF). These kinases phosphorylate and activate MEK1/2 (MAPK/ERK kinase). MEK1/2 then phosphorylates the final kinases in the cascade, ERK1/2, which move into the nucleus to regulate transcription factors (69). ERK signaling functions include cell proliferation by promoting cyclin expression and suppressing cell cycle inhibitors, cell differentiation and migration, cell metabolism and survival (16).

Aside from its role in tumor cells, the MAPK/ERK pathway also influences the TME, which, in turn, impacts tumor growth and therapeutic responses (70). Raf/MAPK/ERK signaling in the TME plays major roles in immune modulation and angiogenesis (71). The ERK signaling pathway plays a key role in controlling T cell activation, differentiation, and cytokine production, all of which are crucial for mounting effective antitumor immune responses (72). When ERK signaling becomes dysregulated, it can lead to T cell dysfunction or exhaustion, ultimately weakening the immune system’s ability to detect and fight cancer (72). In macrophages, dysregulation of the ERK pathway promotes a shift toward the immunosuppressive M2 phenotype, which facilitates tumor growth (73). Similarly, ERK dysregulation supports MDSC expansion and enhances their immunosuppressive activity (74). ERK dysregulation contributes to angiogenesis by promoting endothelial cell proliferation and the formation of new blood vessels, processes that are critical for supporting tumor growth and metastasis (75, 76). Furthermore, in the TME, ERK signaling dysregulation facilitates the secretion of growth factors such as TGF-β and VEGF, as well as enzymes involved in ECM remodeling, thereby promoting tumor invasion and therapeutic resistance (77–79).

Raf/MAPK/ERK signaling normally regulates cell division and differentiation, but when driven by KRAS mutation, it enhances the proliferative and survival capabilities of pancreatic cells (80). In PDAC, persistent activation of Raf/MAPK/ERK results in upregulation of key cell cycle regulators, such as cyclins and cyclin-dependent kinases (CDKs) (81). Additionally, the pro-apoptotic pathway is inhibited, and genes responsible for epithelial-mesenchymal transition (EMT) are activated, thereby supporting invasiveness and metastatic capabilities. This leads early lesions to progress to an aggressive form of cancer (82, 83).

Inhibitors of the Raf/MAPK/ERK pathway play a crucial role in targeting the TME in PDAC. MEK inhibitors such as Cobimetinib, Trametinib, Binimetinib, and Selumetinib can block MEK1/2 thereby preventing ERK activation (84). In the TME, MEK inhibitors can reprogram CAFs, in order to reduce fibrosis and enhance drug delivery (85). Moreover, MEK inhibitors may enhance immune checkpoint therapy, such as anti-PD-1/PD-L1, and enhance antigen presentation by dendritic cells (86, 87). These inhibitors have shown modest activity in PDAC TME. Although BRAF inhibitors such as Vemurafenib and Dabrafenib are effective in BRAF V600E mutants in melanoma and colorectal cancer, they are often ineffective in KRAS mutant tumors, such as in PDAC, due to either resistance mechanisms or paradoxical activation (88–90). Pan-RAF Inhibitors, LY3009120, RAF265, inhibit all RAF isoforms, including BRAF, in order to avoid the paradoxical activation seen with selective BRAF inhibitors (91, 92). ERK inhibitors, such as Ulixertinib (BVD-523) and LY3214996, target the final effector of the cascade and can overcome resistance to upstream inhibitors (92, 93). Since Raf/MAPK/ERK pathway regulates angiogenesis, the inhibition can reduce tumor vascularization. The use of inhibitors alone has proven largely ineffective in treating PDAC due to KRAS mutations, dense fibrotic stroma, and an immunosuppressive TME. As a result, combination therapies are being actively explored (94, 95). Current clinical trials are investigating various strategies, including MEK/ERK inhibitors such as nivolumab combined with immunotherapies, MEK inhibitors combined with anti-fibrotic agents (e.g., PEGPH20), and pan-RAF inhibitors combined with EGFR inhibitors in KRAS-mutant tumors (95–99). Additional combinations currently under investigation include MEK inhibitors with immune checkpoint blockade, ERK inhibitors with autophagy inhibitors, and MEK or ERK inhibitors combined with FAK inhibitors to target and remodel the tumor stroma (100–104).

Despite research on Raf/MAPK/ERK signaling pathway inhibitors, targeting this pathway remains difficult due to the intricate signaling network and the dense TME. Polymeric nanoparticles offer a promising solution by enabling targeted delivery of therapeutic agents, thereby increasing treatment specificity and effectiveness while reducing systemic toxicity (105). Also, polymeric nanoparticles offer a promising approach to improving drug delivery and PDAC treatment outcomes by addressing the challenges posed by the TME and tumor heterogeneity (106). Studies have shown that inhibiting the MAPK pathway can sensitize PDAC cells to autophagy inhibitors, leading to enhanced cell death. Nanoparticle formulations that deliver both MAPK and autophagy inhibitors have shown encouraging results in preclinical studies, suggesting a promising treatment approach for PDAC (107, 108). A key study reported hexadentate PLGA nanoparticles covalently conjugated to PD98059, enabling prolonged intracellular delivery of this MEK1/2 inhibitor. *In vitro*, the nanoparticles significantly reduced ERK phosphorylation and suppressed tumor cell proliferation. *In vivo*, murine studies demonstrated that delivering PD98059-loaded PGM nanoparticles significantly enhanced its pharmacokinetics and biodistribution following intravenous injection, compared with administering soluble PD98059 (109). These findings underscore the promise of polymer-based platforms for targeted inhibition of the MAPK pathway. Furthermore, liposomal curcumin combined with gemcitabine suppressed mTOR signaling, reduced autophagy, and improved treatment outcomes in PDAC (110). A recent patent describes pH-sensitive PEG-DB polymer nanoparticles encapsulating SCH772984, an ERK pathway inhibitor. In MIA PaCa-2 pancreatic cancer cells, these nanoparticles effectively blocked phosphorylated ERK and its downstream RSK signaling, maintaining ERK inhibition longer and performing better than the free drug (111). While still in the preclinical phase, this approach demonstrates the advantages of nanoscale drug delivery and the benefits of pH-triggered drug release. Though primarily targeting upstream regulators, KRAS-silencing nanoparticles have demonstrated downstream inhibition within the MAPK cascade. Using polymer hybrid nanoparticles to deliver KRAS-targeted siRNA reduced ERK activation and significantly impaired tumor cell proliferation in PDAC models. This experiment showed that cRGD-BCP-siKRAS significantly enhanced siKRAS uptake in PANC-1 tumors and achieved 90% knockdown of KRAS G12D. This led to strong tumor suppression and remarkable survival benefits, with a median survival of 101 days compared with 38 days in the PBS group and 59 days in the BCP-siKRAS-only group. Notably, 40% of the treated mice experienced complete tumor regression (112). This approach illustrates the potential of RNAi-based nanocarriers to achieve multi-level suppression of the MAPK pathway through precise genetic modulation.

## TGF-β signaling

The transforming growth factor-beta (TGF-β) signaling pathway plays a paradoxical, context-specific role in PDAC biology (113). TGF-β primarily functions as a tumor suppressor in normal tissues during the early stages of tumor development, inhibiting cell proliferation and inducing apoptosis. Its role is often reversed in advanced PDAC. At the advanced stage, TGF-β commonly supports tumor progression by enhancing invasion, immune evasion, and metastasis (114). This functional transition is a hallmark of PDAC pathogenesis, driven largely by mutations in key downstream effectors and changes within the TME.

The TGF-β pathway can be initiated canonically or in a non-canonical fashion (115). In canonical signaling, the TGF-β signaling pathway begins when TGF-β ligands (TGF-β1, TGF-β2, and TGF-β3) bind to the type II receptor (TGFBR2). This binding triggers the recruitment and phosphorylation (activation) of the type I receptor (TGFBR1). The activated receptor complex then phosphorylates SMAD2 and SMAD3 proteins (Figure 1), which then form a complex with SMAD4, a key mediator. The resultant complex enters the nucleus and regulates the expression of target genes (116, 117). In PDAC, the tumor suppressor gene SMAD4, located on chromosome 18q21, is deleted or mutated in around 50%–55% of cases. This loss is linked to poor patient outcomes, increased risk of metastasis, and resistance to treatment (118). Without functional SMAD4, the canonical TGF-β pathway is disrupted, potentially shifting the signaling balance toward non-canonical, or SMAD-independent, pathways. These alternative routes, such as PI3K/AKT, Rho-like GTPases, MAPK, and NF-κB signaling, can independently promote tumor cell growth, survival, and invasion (119).

In early pancreatic lesions, such as PanIN, the TGF-β pathway plays a protective role by maintaining tissue homeostasis. It does this by enforcing cell cycle arrest via cyclin-dependent kinase inhibitors such as p21 and p15, and by promoting apoptosis (120). However, as PDAC progresses and genetic mutations accumulate, particularly the loss of SMAD4, TGF-β signaling undergoes a significant shift. It transitions from a tumor suppressor to a tumor promoter. This change drives cancer progression by promoting EMT, a process in which epithelial cells lose their structure and gain migratory, invasive properties (121). EMT driven by TGF-β is a key contributor to metastasis and treatment resistance in PDAC. SMAD4 is frequently inactivated in PDAC. In this context, overexpression of Mucin-1 (MUC1) has been linked to increased TGF-β1 secretion and resistance to cell death, which promotes tumor invasiveness through a SMAD4-independent pathway, which may be due to the interaction between MUC1’s cytoplasmic tail and c-Src (122). These findings suggest that MUC1 could be a useful biomarker for identifying patients with SMAD4-deficient PDAC who might respond well to anti–TGF-β treatments.

Besides its direct impact on tumor cells, TGF-β also plays a key role in shaping the immune system and stromal landscape of PDAC. Within the dense stroma of PDAC’s tumor microenvironment, TGF-β plays a crucial role in creating a tumor-promoting environment by interacting with both stromal and immune cells (123). It activates fibroblasts and pancreatic stellate cells (PSCs), encouraging their transformation into CAFs. These CAFs produce large amounts of ECM proteins such as collagen and fibronectin, contributing to fibrotic desmoplasia and forming a physical barrier against therapeutic delivery. TGF-β also regulates the phenotypic balance between inflammatory CAFs (iCAFs) and myofibroblastic CAFs (myoCAFs), primarily by downregulating IL-1R1. This change promotes the formation of a dense, ECM-rich stroma that suppresses immune responses, fuels tumor growth, and hinders the efficacy of immunotherapy (124–126). TGF-β signaling in PSCs further drives fibrosis by increasing collagen deposition and tissue stiffness, factors that not only promote tumor growth but also restrict drug penetration (127). Additionally, TGF-β triggers signaling via ROS and NOX4, helping sustain fibrosis by activating pathways such as MAPK and NF-κB (128). Beyond its fibrotic effects, TGF-β also plays a crucial role in suppressing the immune response. It inhibits cytotoxic T lymphocyte activity by downregulating essential effector molecules such as IFN-γ and granzyme B, promotes the expansion of Tregs, and prevents dendritic cell maturation, thereby reducing antigen presentation (129–131). TGF-β also skews tumor-associated macrophages (TAMs) toward an M2-like immunosuppressive phenotype, enhances PD-L1 expression, and facilitates EMT through SMAD-Snail signaling (132–134). Moreover, TGF-β activity is modulated by environmental factors, such as hypoxia, nutrient deprivation, and mechanical stress, which interact synergistically with the pathway to intensify fibrosis and immune suppression within the PDAC TME. The influence of this pathway on PDAC progression makes it a crucial target.

Therapeutic targeting of TGF-β in PDAC has been achieved using inhibitors, such as Galunisertib (LY2157299) (135, 136), a TGF-βRI kinase inhibitor evaluated in clinical trials for PDAC. Fresolimumab (SAR439459) (137), a pan–TGF-β- neutralizing antibody, and Bintrafusp alfa (M7824), a bifunctional fusion protein targeting both PD-L1 and TGF-β, have also shown promising results in early-phase studies for other cancers and, potentially, for PDAC (138, 139). Combination approaches targeting TGF-β signaling are currently under investigation. These include synergistic strategies with immune checkpoint inhibitors, where TGF-β inhibition reprograms the TME to enhance the effectiveness of anti-PD-1/PD-1/PD-L1 therapies (140, 141). Additionally, a phase III clinical trial has shown that combining TGF-β blockade with chemotherapeutic agents, e.g., NIS793 with nab-paclitaxel and gemcitabine, has shown promise in metastatic PDAC (142). TGF-β inhibition may also sensitize tumors to radiotherapy by enhancing stromal modulation (143).

While TGF-β inhibitors are under investigation, systemic inhibition remains challenging due to their widespread physiological roles and associated toxicity (51). Also, the clinical efficacy of combination therapy for PDAC is very limited. Therefore, there is a critical need for targeted delivery strategies that can selectively modulate TGF-β signaling activity within the TME, enhance drug delivery, and reduce systemic toxicity. Novel delivery approaches involve using nanoparticles to selectively target stromal areas with TGF-β inhibitors (105). This led to decreased stromal fibrosis, increased infiltration of CD8^+^ T cells, and reprogramming of CAFs to a more quiescent state. When combined with gemcitabine or immune checkpoint inhibitors, the nanoparticles significantly enhanced tumor suppression and survival compared with single-agent treatments (144). With this approach, systemic toxicity remained minimal. In a study, biodegradable polymeric nanoparticles (e.g., PLGA-PEG) were designed to encapsulate small molecules, such as TGF-β receptor inhibitors and TGF-β-targeting siRNAs (145). These nanoparticles were functionalized with surface ligands to specifically target CAFs and immune cells within the PDAC TME (146). Their release profiles were engineered to respond to tumor-specific cues, such as acidic pH or elevated reactive oxygen species. *In vitro* and *in vivo* studies using orthotopic PDAC mouse models were performed to assess biodistribution, modulation of the TME, immune activation, and overall therapeutic efficacy (147, 148). Polymeric nanoparticles offer a promising way to specifically inhibit TGF-β signaling within the PDAC tumor microenvironment. This targeted approach helps reduce side effects and boost the effectiveness of existing chemotherapy and immunotherapy treatments. Therapeutically targeting the TGF-β pathway using polymer-based nanoparticles offers a promising strategy to simultaneously remodel the tumor stroma and enhance anti-tumor immune responses. Zhao et al., in a study, developed size-switchable nanoplatforms (NS-TAX@Lipo-VAC) consisting of paclitaxel-loaded nanospheres encapsulated within liposomes containing vactosertib, a TGF-βRI kinase inhibitor (149). In an orthotopic PANC-1 PDAC model, these nanoparticles effectively accumulated in both the tumor core and stroma, enabling deep tissue penetration, reduced ECM deposition, and marked tumor suppression. Li and colleagues engineered MMP-2–responsive alternating copolymer nanoparticles for co-delivery of LY2109761, a TGF-β receptor inhibitor and CPI-613, a mitochondrial metabolism disruptor (150). Within the TME, the MMP-2–sensitive linker was cleaved, releasing CPI-613 from the polymer-peptide complex. This cleavage also caused the nanopolyplex to break apart, releasing LY2109761. Once released, LY2109761 targeted the tumor stroma, disrupting communication between tumor cells and pancreatic stellate cells, while CPI-613 triggered apoptosis in the cancer cells. This dual-targeting approach blocked TGF-β–mediated stromal production and significantly inhibited tumor growth in PDAC xenograft models.

Furthermore, a macrophage-mimetic nanoparticle system was designed by encapsulating SD-208 (a TGF-βRI inhibitor) in PLGA nanoparticles cloaked with macrophage membranes. These biomimetic polymeric nanoparticles selectively targeted TAMs and tumor cells, inhibited TGF-β–driven EMT and M2 macrophage polarization, while enhancing cytotoxic T lymphocyte (CTL) infiltration, suppressing metastasis, and promoting immune activation (151). Another innovative approach involved nanoscale liposomal gels (nLGs) co-delivering vactosertib and IL-2 (145). In Murine PDAC models, this platform successfully modulated the TME by inhibiting TGF-β signaling, increasing infiltration of natural killer (NK) cells and CD8^+^ T cells, and significantly delaying tumor progression. Furthermore, Wang et al. introduced a pH-responsive clustered nanoparticle system (iCluster) for co-delivery of PD-L1 siRNA and LY2157299 (152). Upon exposure to the acidic TME, the nanoparticles released the TGF-β receptor inhibitor, which reduced stromal density, while concurrent PD-L1 silencing enhanced antitumor immunity, demonstrating a synergistic stroma-immune modulatory effect. Despite several clinical trials against important signaling pathways including KRAS, PI3K/AKT/mTOR and MAPK, there has been limitation in their translation (Table 1). To advance clinical application in PDAC, future research should focus on testing therapeutic effectiveness in orthotopic PDAC models that closely mimic the pancreatic tumor microenvironment. Important next steps include optimizing drug pharmacokinetics and carefully assessing systemic toxicity.

**TABLE 1:** Emerging targeted therapies and clinical trials against signaling pathways in PDAC.

Target class	Mechanism of action	Clinical trials, model and phases	Key findings	Limitations in clinical application	Ref.
KRAS inhibitors	Direct inhibition of mutant KRAS (G12D, pan-RAS)	GFH375 first-in-human study; VS-7375; RMC-6236 (RASOLUTE-302) - phase I/IIa	Early KRAS G12D inhibitors show ORR ∼41% and DCR ∼97% in selected cohorts	Mutation-specific applicability; rapid resistance via MAPK/PI3K reactivation; toxicity; incomplete suppression	(153–155)
MAPK pathway inhibitors	Block RAF–MEK–ERK signaling downstream of KRAS	In patients with advanced PDAC- multiple MEK inhibitor combinations - phase I/II trial	Limited monotherapy efficacy; improved response in combinations	Feedback activation; redundancy; poor durability; toxicity in combinations	(156, 157)
PI3K/AKT/mTOR inhibitors	Block survival/metabolic signaling	Advanced cancer patient-NCT00936872 and basket trials phase I trial	Modest efficacy; potential synergy in combinations	Compensatory signaling; narrow therapeutic window; lack of biomarkers	(158)
Immunotherapy (checkpoint and vaccines)	Reactivates anti-tumor immunity; KRAS neoantigen targeting	First-in-human, multicenter, - ongoing phase 1 NCT06105021.KRAS vaccine + ipilimumab + nivolumab trial - phase 1	KRAS-specific T-cell responses observed; early immune activation	Immunosuppressive TME; low TMB; limited response rates; irAEs	(159, 160)
Adoptive T-cell therapy	Engineered TCR-T cells targeting KRAS mutations	First-in-human - ongoing phase 1 NCT06105021	Promising early-phase personalized responses	Complex manufacturing; scalability; patient specificity	(159)
Nanoparticle-based delivery	Enhances drug penetration through dense stroma	Locally advanced pancreatic cancer patient - MSKCC siRNA implant studies - phase II trial	Improved drug delivery and survival trends in early studies	Heterogeneous tumor penetration; manufacturing; safety concerns	(161)
RNA-based therapies (siRNA)	KRAS gene silencing	In human- MSKCC siRNA implant studies NCT01676259 - phase II trial	Feasible early efficacy signals	Stability; delivery efficiency; off-target effects	(161)
Combination therapies	Hedgehog pathway inhibitor (NLM-001) and a CTLA-4 blocker (zalifrelimab)	In human- NCT04827953 -gemcitabine plus nab paclitaxel plus NLM plus Zalifrelimab- phase Ib/IIa	Improved responses. Better PFS and OS	Adverse safety profile and small sample size (28 patients)	(162)
TME-modulating strategies	Targets stroma and immune suppression	PEGPH20 trials (NCT01839487)	PEGPH20 + chemotherapy improves PFS, especially in HA-high pancreatic tumors	Dense desmoplasia; inconsistent translation; No OS benefit, safety concerns, biomarker complexity	(163, 164)

## NF-κB signaling

The nuclear factor kappa-light-chain-enhancer of activated B cells (NF-κB) is a family of transcription factors that regulate genes involved in inflammation, immune responses, cell proliferation, and survival (165). NF-κB plays a central role in cellular responses to various stimuli, including cytokines, stress signals, pathogens, and DNA damage (Figure 1). The family comprises five subunits: RelA (p65), RelB, c-Rel, p50, and p52, which form homo- or heterodimers (166). These dimers are typically kept inactive in the cytoplasm by inhibitor proteins known as IκBs (Inhibitors of κB). Upon stimulation, IκBs are phosphorylated by the IκB kinase (IKK) complex, leading to their ubiquitination and degradation. This allows NF-κB dimers to enter the nucleus and initiate transcription of target genes (167). The activation of NF-κB can occur via either the canonical pathway, triggered by pro-inflammatory signals such as TNF-α and IL-1β, or the non-canonical pathway, which is activated by developmental cytokines such as CD40L and B-cell activating factor (BAFF). While the canonical pathway primarily involves the RelA:p50 dimer and the IKKβ subunit, the non-canonical pathway relies on the RelB:p52 dimer and IKKα (168, 169).

In PDAC, persistent NF-κB activation is observed in both tumor cells and the surrounding stromal components (170). In tumor cells, NF-κB drives the expression of anti-apoptotic genes such as Bcl-xL, Bcl-2, and XIAP, promoting survival and resistance to chemotherapy agents, e.g., gemcitabine (171, 172). In the TME, NF-κB signaling in CAFs and TAMs promotes a fibrotic, pro-inflammatory, immunosuppressive milieu that hinders cytotoxic T cell infiltration and supports tumor progression. Activated NF-κB enhances MDSC activity by inducing the production of colony-stimulating factors like GM-CSF and promoting the secretion of chemokines such as CXCL1, CXCL2, and CXCL5, which attract CXCR2^+^ MDSCs to the TME (173, 174). Additionally, NF-κB activity in the TME enhances the production of cytokines such as IL-6, CXCL12, ECM-degrading enzymes (e.g., MMPs), and VEGF, all of which contribute to tumor growth and metastasis (175). Furthermore, stromal NF-κB signaling promotes immune evasion by driving macrophage polarization toward an M2 phenotype and inhibiting dendritic cell maturation (176).

Multiple categories of NF-κB inhibitors have been investigated for targeting the TME in PDAC. These include: (1) IκB Kinase (IKK) Inhibitors: These agents block the phosphorylation of IκB, thereby preventing NF-κB from translocating to the nucleus (177). BAY 11-7082 is an irreversible inhibitor of IKKβ and has been shown to reduce PDAC tumor growth and overcome stroma-mediated resistance (178, 179). Similarly, BMS-345541 is a selective IKKβ inhibitor that has demonstrated efficacy in murine PDAC models (180). (2) Proteasome Inhibitors: these compounds prevent the degradation of IκB by inhibiting proteasome activity, thereby blocking NF-κB activation (181). Bortezomib, a clinically approved multiple myeloma drug, represents this class. It has been shown to sensitize PDAC cells to chemotherapy, although its use in solid tumors is limited due to systemic toxicity (182). (3) Natural Compounds: Several naturally derived bioactive agents possess anti-NF-κB activity. For instance, curcumin inhibits NF-κB through multiple mechanisms and has been shown to reduce fibrosis and cytokine production in the TME (183, 184). Parthenolide, another natural compound, inhibits IKK activity and exhibits pro-apoptotic effects in PDAC cells. Parthenolide primarily blocks IκB activation, thereby indirectly preventing NF-κB activation and its release from the cytoplasmic IκB complex, and secondarily directly binds NF-κB, inhibiting its ability to interact with DNA (185–187). (4) Nucleic Acid-Based Inhibitors, such as siRNAs and antisense oligonucleotides, are being explored to target key players in the NF-κB pathway, including p65 (RelA), MyD88, and IKKβ. These agents may reduce NF-κB activity in tumor cells and in surrounding stromal cells (188, 189). However, research in this area, particularly for pancreatic cancer, is still in its early stages. Most studies so far have been limited to *in vitro* experiments, with very few moving into animal models or clinical trials. Given NF-κB’s central role in driving tumor progression and inflammation in PDAC, thorough evaluation, especially *in vivo*, is needed to fully understand the therapeutic potential of NF-κB inhibitors such as parthenolide. One promising direction is the use of nanoparticles to deliver these inhibitors. This approach could help address major limitations of systemic therapies, such as low bioavailability and unintended toxicity, by enabling more targeted and efficient delivery to the tumor site. Polymer-based nanoparticles offer a promising approach to improve the delivery and precision of NF-κB-targeted therapies, enhancing tumor specificity while minimizing side effects. Curcumin, a natural inhibitor of NF-κB, has shown therapeutic potential but is limited by its poor bioavailability. Encapsulating curcumin in polymeric nanoparticles known as NanoCurc greatly enhances its pharmacokinetics and effectiveness. In both subcutaneous and orthotopic PDAC models, NanoCurc successfully blocked NF-κB signaling, as evidenced by reduced nuclear translocation of the p65 subunit and lower expression of downstream targets, including MMP-9 and cyclin D1, ultimately resulting in decreased tumor growth and metastasis (190). Similarly, celastrol, a potent NF-κB inhibitor, has faced challenges due to its low solubility, poor bioavailability, and toxicity (191). In recent years, researchers have explored a range of strategies to improve its delivery. Nanotechnology-based formulations, in particular, have shown strong potential to enhance celastrol’s pharmacological efficacy while reducing its side effects (192).

In a study, self-assembling polymeric nanoparticles engineered from elastin-like recombinamers (ELRs), were designed to deliver a small peptide inhibitor targeting the protein kinase Akt. These nanoparticles were tested in both PANC-1 cells and patient-derived xenograft (PDX) models of pancreatic cancer. The ELR-based nanoparticles successfully inhibited Akt phosphorylation and downstream signaling, suppressed NF-κB signaling, and triggered caspase-3-driven cell death. Animal studies showed that these nanoparticles remained in circulation longer and caused minimal toxicity, highlighting their promise as effective drug-delivery vehicles (60). Despite recent advances, nanoparticle-based treatments for PDAC have seen limited clinical translation, largely due to insufficient *in vivo* data. Future research should prioritize validation in immunocompetent PDAC models to assess the impact of NF-κB-targeted polymer nanoparticles. Additional focus should be placed on pharmacokinetic and toxicology profiling, as well as on developing combination therapies that integrate NF-κB-targeted nanoparticles with conventional chemotherapy or immune checkpoint inhibitors.

## JAK/STAT pathway

**FIGURE 2:**
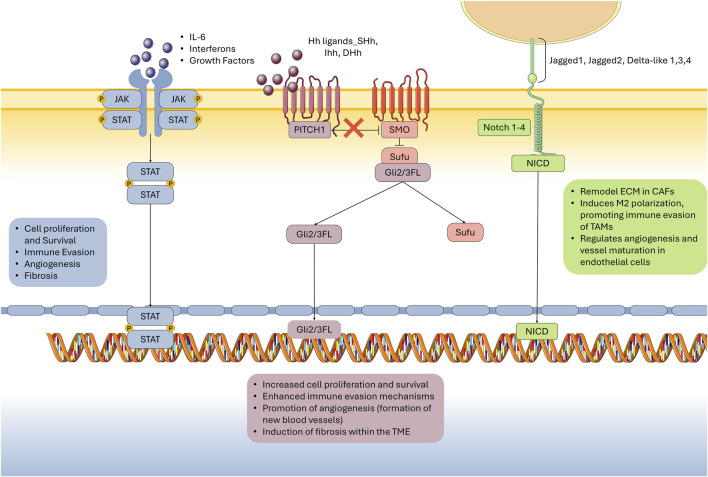
Key developmental and inflammatory signaling pathways involved in PDAC TME. The JAK/STAT pathway is commonly activated by cytokines such as IL-6, leading to phosphorylation and nuclear translocation of STAT proteins, which regulate genes involved in inflammation, immune suppression, and tumor cell survival. The Hedgehog pathway, initiated by ligands like Sonic Hedgehog (SHH), signals through the PTCH-SMO-GLI axis and plays a central role in stromal activation, fibrosis, and maintenance of the TME. The Notch pathway is activated by ligand-receptor interactions between neighboring cells, leading to cleavage of the Notch intracellular domain (NICD) and transcriptional activation of target genes that regulate cell fate, proliferation, and stemness. Dysregulation of these pathways contributes to PDAC tumor growth, stromal remodeling, and resistance to conventional therapies.

The Janus kinase/signal transducer and activator of transcription (JAK/STAT) pathway is a critical signaling axis that regulates a wide array of cellular functions, including proliferation, differentiation, survival, and immune regulation (193). This JAK/STAT cascade is initiated when extracellular cytokines, growth factors or interferons bind to their corresponding membrane-bound receptors. Ligand binding causes the receptor dimerization, which activates the JAKs, a family of enzymes including JAK1, JAK2, JAK3, and TYK2. When activated, JAKs phosphorylate specific tyrosine on the inside of the receptor, creating binding sites that allow STAT proteins to attach (Figure 2). Once recruited, STATs are phosphorylated by JAKs, facilitating their dimerization via SH2-phosphotyrosine interactions. These STAT dimers then translocate to the nucleus, where they bind to DNA response elements and drive the transcription of genes involved in inflammation, cell cycle control, apoptosis resistance, and immune modulation (193–195).

In PDAC, abnormal activation of the JAK/STAT pathway has become a key driver of tumor growth and an immunosuppressive tumor environment (196, 197). The PDAC TME is rich in ligands that activate this pathway, with interleukin-6 (IL-6) among the most significant. Secreted by tumor cells, CAFs, and IL-6 bind to its receptor complex (IL-6R and gp130), strongly activating the JAK1/JAK2–STAT3 signaling axis (198). In addition to IL-6, other molecules such as type I and II interferons (IFNs), granulocyte-macrophage colony-stimulating factor (GM-CSF), epidermal growth factor (EGF), and vascular endothelial growth factor (VEGF) also contribute to sustained JAK/STAT activation within the tumor milieu (199). Collectively, these signals establish a chronically inflamed microenvironment that promotes tumorigenesis while impairing anti-tumor immunity (200).

Constitutive STAT3 signaling supports multiple oncogenic processes in PDAC. STAT3 promotes the expression of genes such as cyclin D1, Bcl-XL, and survivin, which drive unchecked proliferation and resistance to apoptosis (201, 202). Simultaneously, STAT3 lowers the levels of MHC class I and II molecules while increasing the production of immunosuppressive cytokines, such as IL-10 and TGF-β (203). This double effect impairs the body’s antigen-presenting capacity and promotes the accumulation of immunosuppressive cells, such as Tregs and MDSCs, which together suppress the immune response. Furthermore, STAT3 blocks dendritic cell maturation and antigen presentation, weakening T cell activation and the overall adaptive immune response (204). The JAK/STAT pathway also promotes neovascularization by increasing levels of VEGF and other pro-angiogenic factors, helping tumors expand even in low-oxygen conditions (205, 206). Beyond its effects on the immune system, JAK/STAT signaling also influences the tumor stroma, CAFs. Cytokines such as IL-6 and IL-1β activate these fibroblasts via STAT3, promoting the production of ECM proteins, including collagen and fibronectin. This remodeling of the ECM not only forms a physical barrier that blocks immune cells and reduces drug delivery but also supports further tumor growth (207). Additionally, STAT3 activation in CAFs stimulates the production of MMPs, enzymes that degrade ECM components and promote tumor invasion and metastasis (208). Overall, the JAK/STAT pathway is deeply involved in shaping the PDAC TME, playing a central role in immune evasion, stromal changes, and chronic inflammation. These findings underscore the potential of targeting JAK/STAT signaling to reprogram the TME and enhance the effectiveness of both conventional treatments and immunotherapies.

Targeting the JAK/STAT signaling pathway offers a promising therapeutic approach in PDAC, aiming to reduce inflammation and immune suppression and to disrupt harmful interactions within the tumor stroma. JAK inhibitors such as ruxolitinib, along with STAT3 inhibitors such as napabucasin and AZD1480, have demonstrated varying levels of preclinical and clinical activity (209). Although ruxolitinib did not meet endpoints in Phase III trials, it may offer benefit for IL-6–high PDAC subsets when combined with other therapies. Preclinical studies using PDAC cell lines and animal models showed that napabucasin could suppress tumor growth and metastasis. It worked by blocking STAT3 phosphorylation and reducing spheroid formation, both of which are often used to model cancer cell proliferation. While these early results were promising, clinical trials ultimately failed to demonstrate significant benefits, leading to the discontinuation of napabucasin’s trial (210, 211). Napabucasin has shown potential in targeting cancer stem cells (CSCs) despite the halted trial. Another compound, AZD1480, a JAK2 inhibitor, also prevents STAT3 activation by blocking phosphorylation at tyrosine 705 (Y705), a key step in the signaling process. It has been shown to lower the expression of STAT3-regulated genes, including those that control cell division and prevent apoptosis. Both *in vitro* and *in vivo* studies have supported its effectiveness. It inhibits STAT3 activation and its downstream signaling pathways, reduces cell proliferation, promotes apoptosis, and may help overcome chemoresistance in PDAC (212, 213).

Monotherapies targeting the JAK/STAT pathway have been largely ineffective in PDAC, primarily because of the complex and immunosuppressive nature of its TME (214). Consequently, several rational combination strategies are being explored. These include combinations with immunotherapy, chemotherapy, stromal modulators, and IL-6 blockade to help overcome TME-mediated resistance. For instance, JAK/STAT inhibitors, when combined with checkpoint inhibitors, can reinvigorate T cells and boost immune response. A phase I clinical trial evaluated the combination of ruxolitinib and the anti–PD-1 antibody nivolumab in patients with Hodgkin lymphoma who were relapsed or refractory after prior checkpoint inhibitor therapy. The combination achieved an overall response in 10 out of 19 patients. Ruxolitinib significantly lowered the neutrophil-to-lymphocyte ratio and reduced MDSC levels, while boosting the number of cytokine-producing T cells. Additionally, ruxolitinib restored the function of exhausted T cells and enhanced the efficacy of immune checkpoint blockade in preclinical models of solid tumors and lymphoma (215). Also, combining JAK/STAT inhibitors with IL-6/IL-6R blockade can suppress upstream JAK/STAT signaling. Combining JAK inhibitors with IL-6 or IL-6 receptor (IL-6R) blockers offers a more layered approach to suppressing the signaling pathway. While JAK inhibitors directly target the kinases involved, blocking IL-6 or its receptor prevents the pathway’s initiation by this key inflammatory cytokine (216, 217). Together, this dual-targeted strategy can provide stronger and longer-lasting inflammation suppression.

Looking ahead, future strategies may involve precision medicine approaches, including nanoparticle-based delivery systems to target non-canonical STAT3 functions more effectively. For instance, when stroma targeting agents are combined with JAK/STAT inhibitors, this can disrupt fibroblast-tumor-immune cell networks (218). While STAT3 remains a promising target for cancer therapy, developing effective small-molecule inhibitors has been limited by challenges such as poor solubility, off-target toxicity, and insufficient tumor accumulation. To overcome these challenges, polymer-based nanoparticle systems have gained attention as effective delivery platforms. They offer targeted delivery, controlled release, and the ability to co-deliver other treatments, such as chemotherapy or RNA-based drugs, for enhanced therapeutic impact. An example is curcumin, a natural STAT3 inhibitor, which has been formulated into biodegradable polymeric nanoparticles to improve its bioavailability and therapeutic effectiveness. In a study by Mohanty and Mukherjee, this formulation, known as NanoCurc, significantly reduced STAT3 phosphorylation, IL-6 production, and angiogenesis, ultimately leading to decreased tumor growth and metastasis in orthotopic PDAC models (190). A recent report showed that co-delivery of WP1066, a small-molecule JAK/STAT pathway inhibitor, and STAT3 siRNA encapsulated within these targeted liposomes resulted in significant suppression of orthotopic glioblastoma growth in mice, exceeding 350% inhibition compared to the untreated control group (219). Additionally, Fong et al. showed that encapsulating the STAT3 inhibitor Stattic within chitosan-coated PLGA nanoparticles (C-PLGA) significantly enhanced both *in vitro* and *in vivo* antitumor and antimetastatic effects. These nanoparticles, approximately 142 nm in size, demonstrated improved tumor accumulation via the enhanced permeability and retention (EPR) effect, resulting in over 33% tumor growth inhibition and a marked reduction in metastases in a 4T1 breast cancer model (220). Navarro-Marchal et al. developed CD44-targeted lipid nanocapsules designed to deliver siRNA and chemotherapeutics to pancreatic cancer stem cell (CSC)–rich populations (221). Although not STAT3-specific, this modular system offers a versatile platform adaptable for RNA interference (RNAi) therapies aimed at silencing STAT3 expression in both tumor and stromal compartments. Future research should prioritize refining RNA-loaded nanoparticle systems that target STAT3, with an emphasis on integrating them into well-designed combination therapies. It will also be important to move these approaches into early-phase clinical testing, especially using immunocompetent orthotopic PDAC models that better reflect the complexity of the human disease.

## Hedgehog signaling pathway

The Hedgehog (Hh) signaling pathway is a highly conserved developmental pathway essential for embryonic development, regulating processes such as cell differentiation, tissue organization, and stem cell maintenance (222). In adult tissues, Hedgehog activity is typically low but can be reactivated during tissue repair or in certain diseases, including cancer (223). Increasing attention has been given to its role in fibrotic stroma formation and maintenance. Aberrant activation of the Hedgehog pathway in stromal cells has been linked to ECM deposition, impaired drug delivery, and enhanced tumor progression (223).

The Hedgehog pathway includes key components such as Sonic Hedgehog (SHh), Patched (PTCH), Smoothened (SMO), and the GLI family of transcription factors. In the canonical pathway, Hedgehog ligands regulate gene expression via a receptor-ligand mechanism (224). In the absence of ligands such as SHh, Indian Hedgehog (IHh), or Desert Hedgehog (DHh), the PTCH1 receptor suppresses SMO, thereby preventing downstream signaling. Under these conditions, GLI transcription factors (GLI1–3) are held in the cytoplasm by the Suppressor of Fused (SUFU) protein and processed into repressor forms that inhibit target gene expression. When Hedgehog ligands bind to PTCH1, this inhibition is lifted, enabling SMO to accumulate in the primary cilium and activate the pathway (223). Consequently, GLI proteins dissociate from SUFU, enter the nucleus, and activate transcription of genes involved in cell proliferation, survival, angiogenesis, and EMT (Figure 2).

PDAC is characterized by a dense fibrotic stroma, known as desmoplasia, composed of CAFs, ECM components, and immunosuppressive cells. This TME impairs blood vessel formation, making it harder for systemic treatments to reach the tumor effectively. In PDAC, the Hedgehog signaling pathway plays a central role in driving the development of this dense, fibrotic tissue (225, 226). Tumor cells predominantly secrete SHh, which activates the Hedgehog pathway in neighboring stromal fibroblasts through a paracrine mechanism. That is, rather than signaling within the tumor cells themselves, SHh acts on adjacent stromal cells, leading to their proliferation, fibroblast activation, and excessive ECM production (227, 228). This results in stiff, hypovascular TME that increases interstitial pressure and hinders drug penetration. Preclinical studies have shown that Hedgehog pathway inhibition can reduce stromal density, enhance vascularization, and improve the delivery of chemotherapeutics such as gemcitabine and paclitaxel (229, 230). Several drugs have been developed to block the Hedgehog signaling pathway, including cyclopamine, sonidegib, vismodegib (GDC-0449), and GANT61. Cyclopamine, a natural compound that inhibits the SMO protein, was one of the first to be tested in preclinical studies. Cyclopamine targets and inhibits SMO, a critical component of the Hedgehog pathway, and has demonstrated the ability to suppress cancer cell proliferation and metastasis by inhibiting the proliferation of CSCs (231, 232). While cyclopamine showed encouraging results in early lab studies, its progress toward clinical use has faced major setbacks due to significant challenges. Poor solubility which limits effective systemic delivery and severe side effects, particularly teratogenicity, observed in animal models raise safety concerns for human use. Moreover, at concentrations just above those required to inhibit the Hedgehog pathway, cyclopamine may also exert off-target effects on cell growth (233, 234). Because of cyclopamine’s limitations, researchers have explored new derivatives of the drug designed to be safer and more effective in the body (235). While preclinical studies on these derivatives and other SMO inhibitors were promising, clinical trials in PDAC have yielded mixed results, underscoring the difficulty of targeting the Hedgehog pathway in this disease (236). Sonidegib (LDE225) and vismodegib (GDC-449), are examples of cyclopamine derivatives that have both been FDA-approved for the treatment of basal cell carcinoma and have also been evaluated in PDAC (237, 238). In contrast to SMO inhibitors, GANT61 acts further downstream by blocking GLI transcription factors, which directly shut off the pathway’s gene activity. This makes GANT61 a potential option for tumors that do not respond to drugs targeting the upstream pathway (239).

Although SMO inhibitors showed promise in preclinical PDAC studies, the results from clinical trials have been mixed. Some findings indicated that complete stromal depletion via Hedgehog pathway inhibition might inadvertently promote tumor progression, possibly by removing fibroblasts that help restrain tumor growth or by increasing vascular permeability (240, 241). As a result, the therapeutic strategy has shifted from total stromal ablation to stromal reprogramming, with the goal of restoring a more normalized and functional TME rather than eliminating it entirely (240). Also, challenges such as systemic toxicity and poor tumor targeting have limited clinical efficacy. These issues have led researchers to explore nanoparticle-based delivery systems to improve drug targeting and reduce toxicity (241). For instance, Hedgehog (Hh) pathway inhibitors, such as cyclopamine and GDC-0449, have shown promise, but their clinical impact has been limited by poor delivery and unwanted effects on healthy tissues (242). Polymer-based nanoparticles offer a promising alternative by improving pharmacokinetics, directing it more precisely to the tumor, and helping to break down the dense tumor stroma. Zhang et al. had previously developed cyclopamine-loaded PLGA nanoparticles coated with erythrocyte membranes (CMNPs), which improved nanoparticle stability and circulation time (243). In another approach, formulated NanoHHI PLGA-PEG nanoparticles encapsulating the GLI inhibitor HPI-1 were used. In orthotopic Pa03C PDAC xenografts, NanoHHI combined with gemcitabine significantly reduced tumor burden and overcame resistance to SMO inhibition (244). A study utilized mesoporous silica nanoparticles (MSNs) sequentially loaded with cyclopamine (CyP-MSNs) and gemcitabine/cisplatin. This staged delivery strategy effectively depleted the stroma, enhanced perfusion, and improved chemotherapeutic efficacy in HPAF-II PDAC models (245). Additionally, another study described a polymeric prodrug nanoparticle system co-delivering SN38 and GDC-0449. In pancreatic stellate cell (PSC)–enriched PDAC models, this combination inhibited GLI1 signaling, reduced collagen and α-SMA expression, and induced tumor cell apoptosis (246).

Future studies should prioritize evaluation in immunocompetent PDAC models to assess the impact of Hedgehog-targeting nanoparticles, as these models provide more predictive data, especially for evaluating potential immune-related toxicities or synergies with immunotherapies. Also, detailed pharmacokinetic and safety profiling is warranted for targeting SMO and GLI. In addition, combining Hedgehog-targeted nanoparticles with other treatments, such as immune checkpoint inhibitors, stroma-modulating agents, or conventional chemotherapy, could yield even better results.

## Notch signaling pathway

Notch signaling is a highly conserved cell-cell communication pathway that regulates key biological processes such as cell fate determination, proliferation, apoptosis, and differentiation (247). The pathway comprises four receptors (Notch1–4) and five ligands (Jagged1, Jagged2, and Delta-like 1, 3, and 4). Activation occurs through ligand-receptor interactions between adjacent cells, triggering proteolytic cleavage of the receptor and release of the Notch intracellular domain (NICD). The NICD then translocates to the nucleus, where it binds to the CSL (CBF1-Suppressor of Hairless-LAG-1) transcriptional complex to drive the expression of target genes, including members of the HES and HEY families, which influence cell differentiation, proliferation, survival, and apoptosis (248, 249).

In PDAC, abnormal Notch signaling plays a major role in driving tumor growth, supporting CSCs, and shaping the TME (250). Both Notch receptors and their ligands are often overactive in tumor cells and in surrounding supportive cells such as CAFs and TAMs (251). This dysregulation helps maintain the CSC population, promotes EMT via transcription factors such as Snail and Slug, and increases tumor cell resistance to apoptosis (252). Importantly, Notch signaling helps sustain a subgroup of CSCs that are particularly resistant to treatment, which can lead to tumor relapse.

In the TME, Notch signaling plays a central role in intercellular communication. In CAFs, it promotes ECM remodeling and increases the release of tumor-promoting signals, contributing to a stiff, fibrotic, and immunosuppressive environment (253). In immune cells, Notch signaling promotes the polarization of macrophages and other immune subsets toward immunosuppressive phenotypes (254). In endothelial cells, the Notch-Dll4 pathway helps regulate new blood vessel growth, balancing sprouting and vessel maturation, processes that support tumor spread and metastasis (255).

Given its wide-ranging influence in PDAC, Notch signaling is a promising therapeutic target. Several approaches have been explored, including gamma-secretase inhibitors (GSIs) such as MK-0752 and RO4929097, which block a key step in Notch activation by inhibiting γ-secretase-mediated receptor cleavage (256, 257). Preclinical studies have shown that these inhibitors can shrink tumors, reduce CSCs, and slow metastasis (258). However, their use in patients has been limited by gastrointestinal side effects, as Notch also plays a key role in maintaining gut health (259, 260). To address this, alternative dosing strategies and intermittent treatment approaches should be evaluated. Other strategies aim to improve specificity and reduce side effects, such as using monoclonal antibodies that target individual Notch receptors or ligands. For example, antibodies against Notch1 or Jagged1 have shown promise in disrupting CSC niches and tumor-stroma interactions in preclinical models (261, 262). Likewise, anti-Dll4 antibodies have been shown to reduce angiogenesis (263). Other emerging strategies, including ligand-blocking peptides and small molecule inhibitors, are being designed to interfere more precisely with key parts of the Notch pathway, such as blocking interactions between NICD and its downstream partners like CSL or Mastermind-like proteins (264, 265). These novel agents aim to enhance specificity while minimizing toxicity. Given the complexity of the PDAC tumor environment, using Notch-targeted therapies alone may not be enough. Combination treatments are being actively explored, pairing Notch inhibitors with chemotherapy, immune checkpoint inhibitors, or agents that target the tumor stroma, to overcome resistance and enhance the body’s immune response against the cancer (265–267).

Despite promising preclinical data, clinical translation remains challenging. On-target toxicity, tumor heterogeneity, and compensatory signaling pathways limit efficacy. The pleiotropic nature of Notch in normal physiology narrows the therapeutic window (268). Future strategies should emphasize the development of highly selective, context-specific Notch inhibitors, along with reliable biomarkers to help identify which patients are most likely to benefit. Furthermore, since PDAC cells are highly adaptable and can rely on multiple signaling pathways to survive, combination therapies targeting multiple mechanisms will likely be needed. Early-stage clinical trials are already exploring these approaches and may offer valuable insights into overcoming treatment resistance (269). As research progresses, it’s important to better understand the specific roles Notch signaling plays within different parts of the TME and how best to integrate Notch-targeted therapies into broader, multimodal treatment plans. A novel delivery method uses nanoparticle-based drug delivery systems to target components of the Notch pathway more precisely. These approaches aim to enhance treatment effectiveness while minimizing harmful side effects to the rest of the body. In recent years, polymer-based nanoparticles have attracted attention as a means to deliver Notch-modulating therapies directly to tumors, aiming to enhance PDAC’s responsiveness to chemotherapy and immunotherapy. Biodegradable polymers like polylactic acid (PLA) have shown significant potential. For example, researchers have developed PLA nanoparticles loaded with chrysin, a natural flavonoid, to specifically target PDAC CSCs and suppress Notch signaling (270). This approach not only reduced the formation of tumor spheres, which are clusters of aggressive cancer cells, but also made the cancer more responsive to gemcitabine, a commonly used chemotherapy drug. These findings highlight how flavonoid-loaded nanoparticles can deliver both direct anti-tumor effects and disrupt key survival pathways in the TME. Other research has employed polymeric micelles composed of PEG-PLGA copolymers to co-deliver γ-secretase inhibitors (GSIs), such as RO4929097, in combination with standard chemotherapeutics. These micellar formulations can effectively penetrate the dense PDAC stroma and release their therapeutic cargo in a controlled manner, thereby suppressing Notch activation and mitigating CSC-driven resistance (271). Additionally, polyamidoamine (PAMAM) dendrimers have been used to deliver miR-34a mimics, tumor-suppressive microRNAs that downregulate Notch1 and Notch2 expression (272). This approach has successfully reduced the growth and spread of CSCs in both *in vitro* and animal studies, demonstrating that dendrimers have high gene-loading efficiency, target cells specifically, and exhibit minimal toxicity. Altogether, these results demonstrate the important role of polymeric nanocarriers in overcoming the therapeutic challenges posed by PDAC’s complex microenvironment. Targeting the Notch signaling pathway enables these nanoparticles to do more than just block tumor growth; they also degrade the tumor’s supportive environment, enabling more effective combination therapies and potentially enhancing clinical outcomes.

## CXCL12/CXCR4 signaling pathway

**FIGURE 3:**
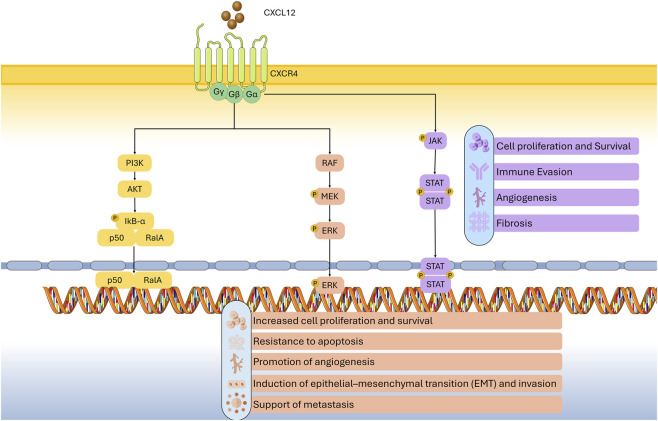
CXCL12/CXCR4 signaling pathway in PDAC TME. CXCL12/CXCR4 axis in PDAC tumor progression and tumor–stroma interactions. CAFs, pancreatic stellate cells (PSCs), endothelial cells, and other stromal components secrete the chemokine CXCL12 which binds to its cognate G protein–coupled receptor CXCR4 expressed on PDAC cells. Ligand binding induces receptor conformational change and activation of heterotrimeric G proteins, leading to downstream signaling through PI3K/AKT, MAPK/ERK, JAK/STAT.

The CXCL12/CXCR4 pathway is among the most evolutionarily conserved and functionally diverse. It is a central regulator of embryogenesis, stem cell trafficking, immune surveillance and pathological processes (273). The CXCL12 gene, also known as stromal cell-derived factor-1 (SDF-1), is located on chromosome 10 (10q11.21) and encodes several variants, including SDF-1α, β, and γ, with differences in stability and tissue distribution (274). On the other hand, CXCR4, also known as CD184, is a seven-transmembrane G-protein-coupled receptor that is mostly located on cell surfaces. They are widely expressed on hematopoietic cells, endothelial cells, nerve cells and many cancer types (275). When CXCL12 binds to CXCR4, CXCR4 undergoes conformational changes in a two-stage process involving the inward rotation of tyrosine 302 (Y302) followed by the outward movement of transmembrane helix 6 (TM6), thus activating its two subunits, Gαi and Gβγ subunits (276). This conformational change triggers the following pathways: PI3K/Akt pathway to promote cell survival, proliferation; central to EMT and metastasis in tumor, the Ras/ERK/MAPK signaling to drive transcriptional activation, cell cycle progression, the ERK phosphorylation which is critical for CXCL12-induced migration in endothelial cells, and the JAK/STAT pathway to influence gene expression in immune and progenitor cells via a G protein–independent mechanism (276, 277). In cancer, this ligand-receptor interaction triggers a signaling cascade within cells that influences cell migration, survival, and proliferation (278). Among the key mediators of the hostile PDAC TME is the CXCL12/CXCR4, largely produced by CAFs. CXCL12/CXCR4 is a key player in orchestrating tumor-stromal-immune interactions that support cancer cells’ evasion of the immune system, metastasis, and therapeutic resistance (279). The CXCL12/CXCR4 signaling pathway interacts with several other oncogenic pathways (Figure 3), such as PI3K/AKT/mTOR, ERK, Hedgehog, JAK/STAT, NF-κB, and mutant p53, through a feedback loop to drive cancer progression (280). The CXCL12/CXCR4 signaling pathway exhibits both reciprocal regulation and feed-forward loops. Reciprocal regulation occurs when CXCL12 and CXCR4 influence each other’s expression activity or level, while feed-forward loops aid the pathway’s signaling intensity over time, potentially increasing its effects (281). In reciprocal regulation, CXCL12 binds to CXCR4, to activate the downstream signaling pathways. This activation can lead to CXCR4 self-upregulation, which creates a positive feedback loop. Additionally, the CXCL12/CXCR4 pathway can trigger other signaling pathways, such as STAT3, which, in turn, can regulate CXCL12 expression. Therefore, the CXCL12/CXCR4 axis and STAT3 pathway can influence each other’s activity in a reciprocal manner (282).

In the feed-forward loops, both amplification and sustained signaling occur. During amplification, a single activation of the CXCL12/CXCR4 pathway can initiate a chain reaction, leading to increased CXCL12 production and/or activation of downstream pathways such as MAPK, which in turn can increase CXCR4 expression. Whereas sustained signaling induces positive feedback, leading to sustained and amplified signaling and potentially prolonging the effects of CXCL12/CXCR4 stimulation (283). These regulatory mechanisms can significantly influence cell behavior, particularly in cancer metastasis and immune cell trafficking. Simply put, the CXCL12/CXCR4 pathway does not just respond to signals; it can also generate signals that amplify its own activity, and it can interact with other pathways in a reciprocal manner. This creates a complex network of interactions that control various cellular processes. Understanding the complexities of these loops and reciprocal regulations is crucial for developing targeted therapies that can effectively modulate the CXCL12/CXCR4 pathway. Several methods for targeting CCL12/CXCR4 have been developed. (1) The use of Peptide mimetics and cyclic peptides that are designed to closely resemble natural molecules, giving them high specificity and strong binding to receptors like CXCR4. They can either mimic the function of CXCL12 or block CXCR4 by inhibiting it competitively (283). An example is CTCE-9908, a CXCL12 analog that inhibits tumor growth and metastasis in preclinical breast and prostate cancer models by competing with endogenous ligand binding to CXCR4 (284, 285). (2) Many small-molecule inhibitors are currently being developed and designed to work better in cancer treatment by offering improved selectivity, effectiveness, and pharmacokinetic profiles. This includes Ulocuplumab (BMS-936564), a human IgG4 monoclonal antibody targeting CXCR4 that blocks CXCR4–CXCL12 signaling. It has shown synergistic effects when combined with chemotherapy in leukemia and lymphoma (286). Also, in a Phase Ib/II trial in relapsed or refractory multiple myeloma, ulocuplumab was well tolerated and showed encouraging activity in combination with lenalidomide and dexamethasone, achieving an overall response rate of 55.2%. In PDAC, CAFs secrete CXCL12, which binds CXCR4 and limits T-cell infiltration into the tumor. By inhibiting CXCR4–CXCL12 interactions, ulocuplumab may help restore immune cell access to the tumor microenvironment, supporting its potential as a therapeutic strategy worth exploring in PDAC (287). Another agent, LY2510924, a cyclic peptide antagonist, has been tested in phase I clinical trials for solid tumors, including pancreatic and colorectal cancers (288, 289). (3) Instead of targeting the receptor directly, some strategies aim to neutralize the CXCL12 ligand to prevent CXCR4 activation (290). (4) Combination therapy is also being explored. For instance, NOX-A12, a CXCL12 inhibitor, is currently being tested in combination with checkpoint inhibitors and radiation therapy. NOX-A12 (olaptesed pegol) is a PEGylated L-RNA aptamer that binds to and captures CXCL12 (290, 291). This approach has shown encouraging results in breaking down the protective environment of other cancers, such as chronic lymphocytic leukemia and glioblastoma, thus enhancing immune infiltration and improving chemotherapy.

The fibrotic nature of PDAC TME supports drug resistance; hence, the use of nanoparticles that are tailored to penetrate this barrier is emerging. In a recent study, LNP-PAMD/siRRM2 nanoparticles preserve the functional properties of AMD3100 (Plerixafor), which blocks CXCR4 to inhibit cell migration and activate the AKT signaling pathway. They further enable the efficient delivery of siRRM2 via a lipid-based shell, thereby markedly enhancing pancreatic cancer cells’ sensitivity to gemcitabine by downregulating RRM2. The LNP-PAMD system also suppresses CXCL12 secretion by activated PSCs, reduces ECM production, and modulates the TME by promoting the recruitment of CD4^+^ and CD8^+^ T cells (292). In another study, Cholesterol-modified polymeric CXCR4 (PCX) antagonist nanoparticles were designed to disrupt cancer-stroma interactions and co-deliver anti-miR-210, to inactivate stroma-producing PSCs along with siKRAS G12D to target pancreatic cancer cells. After intraperitoneal (IP) administration, the nanoparticles preferentially localized to orthotopic syngeneic pancreatic tumors and metastases, which exhibited disrupted mesothelium, thereby enabling effective tumor penetration. These multifunctional nanoparticles modulated the desmoplastic TME by simultaneously blocking CXCR4 signaling and silencing miR-210 and KRAS G12D, leading to the deactivation of PSCs and enhancing cytotoxic T cell infiltration (148). Given the extensive crosstalk between CXCL12/CXCR4 signaling and other pathways involved in PDAC progression, polymeric nanoparticles offer a strategic advantage for targeting multiple signaling pathways within the PDAC TME by modulating the CXCL12/CXCR4 axis.

## Polymer-based nanoparticle precision therapies for PDAC TME

A major challenge in treating PDAC is its dense and complex TME, which not only fuels tumor growth but also hinders drug penetration. To address this, nanomedicine has emerged as a promising approach to improve drug delivery, increase therapeutic precision, and overcome stromal resistance (293, 294). Among the many types of nanocarriers being studied, polymer-based nanoparticles stand out for their structural flexibility; they can be engineered with a wide range of physical and chemical properties for targeted or drug release and stimuli-responsive delivery (295). Various polymer-based nanoparticles have been developed specifically for PDAC therapy (Table 2). The classification is structured by polymer composition, nanostructure, and functionalization mechanisms, each contributing uniquely to the therapeutic system’s overall efficacy. Furthermore, PDAC TME characteristics have been linked to nanoparticle-based therapeutic strategies (Table 3). Although these strategies have been observed to have some delivery barriers limiting their translation.

**TABLE 2:** Polymeric-based nanoparticle strategies targeting key signaling pathways in the cancer TME.

Target pathways	Function in TME	Polymeric nanoparticles used	Preclinical model and mechanism of action	Outcomes	Ref.
PI3K/AKT/mTOR	Cell survival, metabolism, and proliferation	1. PLGA–NPs rapamycin 2. PEG–PCL NPs with dual PI3K/mTOR inhibitors 3. PLA–PEG NPs micelles co-delivering paclitaxel and PI3K inhibitor	MCF-7 and MDA-MB-468. TNBC cell line MDA-MB-231-disrupt AKT/mTOR signaling	Tumor regression, sensitization to chemotherapy	(62, 296)
RAF/MAPK/ERK	Cell proliferation, drug resistance	1. PLGA NPs with MEK inhibitor (e.g., trametinib, paclitaxel) 2. PLGA NPs coated with the macrophage cell membrane and erlotinib3. PLA NPs with dual delivery (MAPK inhibitor + chemo)	KPC in mouse model in the C57BL/6J background. Cre-activated *Kra Trp53* mouse- inhibit MAPK cascade to prevent tumor growth	Enhanced chemotherapy sensitivity, tumor suppression	(297–299)
NF-κB	Inflammation, drug resistance, and cytokine expression	1. Curcumin-loaded PLGA NPs 2. PEI NPs with NF-κB p65 siRNA 3. Hyaluronic acid–coated PLA NPs with IKKβ inhibitor	Breast cancer xenograft in nude mice induced by MDA-MB-435 cells- block nuclear translocation of NF-κB	Reduced inflammatory cytokines, improved drug response	(300–302)
TGF-β	Fibrosis, CAF activation, and immune suppression	1. PEG–b-PCL NPs with TGF-β (LY2157299) siRNA 2. PEI-modified NPs with TGF-β receptor inhibitor 3. PLA-PEG NPs with dual delivery of TGF-β and PD-L1 siRNA	Panc02 xenograft model and an orthotopic tumor model- block TGF-β signaling to reduce fibrosis and immune suppression	Reduced ECM deposition, enhanced immune cell access	(152, 303)
JAK/STAT3	Immunosuppression, tumor growth	1. Chitosan NPs with STAT3 siRNA 2. PEG–PLGA NPs delivering JAK inhibitor, ruxolitinib	MDA-MB-231 and 4T1 cells- silence STAT3 or block JAK kinases -	Reduced MDSCs, improved CD8^+^ T cell function	(220)
Hedgehog (Hh)	Stromal barrier, CAF signaling	1. Biomimetic cyclopamine-loaded PLGA NPs 2. Chitosan-coated nanoparticles with SMO inhibitors 3. PEG–PCL NPs delivering Hh siRNA	Human-derived pancreatic cancer Capan-2 cell line and HUVEC line- inhibit SMO or Gli proteins in Hh pathway	Reduced desmoplasia, increased chemotherapy penetration	(304, 305)
Notch	Pancreatic stem cell renewal, apoptosis, EMT, stromal signaling	1. DLL4-targeted PLGA NPs 2. γ-secretase inhibitor-loaded PEG–PLA NPs 3. Chitosan NPs with Notch1 siRNA	Primary human pancreatic xenografts. HUVEC- block ligand-receptor notch interaction or cleavage	Reduced the percentage of CSCs and tumorsphere formation	(271, 306)
CXCL12/CXCR4	Desmoplastic TME and drug resistance improvement	1. PLGA–AMD3100 NPs 2. PEG-PLGA NPs co-delivering CXCR4 inhibitor and anti–PD-1 3. Chitosan-based NPs with CXCR4 siRNA	Murine- inhibit CXCL12-CXCR4 signaling to enhance T cell infiltration	Reduce the migration and invasion of pancreatic cancer cell	(307, 308)

This table summarizes recent advances in the use of polymeric-based nanoparticles to modulate critical signaling pathways within the PDAC TME.

**TABLE 3:** Linking PDAC TME characteristics to nanoparticle-based therapeutic strategies and delivery barriers.

TME characteristic	Key signaling pathways	Resulting delivery barriers	Nanoparticle-based strategies	Ref.
Dense stromal desmoplasia (CAF activation, ECM deposition)	TGF-β, Hedgehog, KRAS	Increased interstitial fluid pressure; reduced vascular perfusion; poor drug penetration	ECM-degrading nanoparticles (e.g., hyaluronidase-loaded); co-delivery of stromal inhibitors with chemotherapeutics	(309)
Hypoxia and aberrant vasculature	KRAS, PI3K/AKT/mTOR, MAPK	Limited oxygen availability; reduced nanoparticle accumulation; therapy resistance	Hypoxia-responsive nanoparticles; oxygen-generating nanocarriers; vascular normalization via pathway-targeted delivery	(310, 311)
Immunosuppressive microenvironment (TAMs, Tregs, MDSCs)	NF-κB, JAK/STAT3, CXCL12/CXCR4, notch	Immune evasion; poor T-cell infiltration; resistance to immunotherapy	Nanoparticles delivering STAT3/NF-κB inhibitors; CXCR4-targeted systems; co-delivery of checkpoint inhibitors and immunomodulators	(312)
KRAS-driven oncogenic signaling and pathway redundancy	KRAS, MAPK, PI3K/AKT	Intrinsic drug resistance; compensatory signaling; poor therapeutic response	siRNA/miRNA-loaded nanoparticles targeting KRAS; multi-drug nanocarriers for simultaneous pathway inhibition	(313, 314)

## Methods

A structured literature search was performed in PubMed/MEDLINE, Scopus, and Web of Science using predefined keywords covering PDAC signaling pathways, tumor microenvironment components, and polymeric nanomedicine, with most studies published between January 2000 and March 2026 included. Inclusion criteria include limiting the review to peer-reviewed studies with clear mechanistic or therapeutic relevance to PDAC, while excluding non-English articles, abstracts without full text, redacted publications and studies outside the scope. The study selection process includes title and abstract screening, full-text assessment, and removal of duplicates, and is summarized in a PRISMA-style flow diagram (315). In addition, we included a brief quality assessment, evaluating preclinical studies based on experimental rigor and clinical studies using established risk-of-bias principles (316). Together, these revisions strengthen the rigor, reproducibility, and overall transparency of the review.

## Conclusion

The TME of PDAC plays a central role in driving its aggressive biology and therapeutic resistance. Key signaling pathways such as KRAS, PI3K/mTOR/AKT, Raf/AKT/mTOR, JAK/STAT, TGF-β, Hedgehog, NF-κB, Notch and CXCR4/CXCL12 are dysregulated in both cancer cells and stromal components, fostering a pro-tumorigenic milieu marked by desmoplasia, immune evasion, and enhanced metastatic potential. These pathways contribute to complex crosstalk between tumor cells, fibroblasts, immune cells, and extracellular matrix components, reinforcing tumor growth and survival. A deeper understanding of these interconnected signaling networks within the PDAC TME is essential for developing effective, targeted therapeutic strategies that can disrupt this malignant ecosystem and improve patient outcomes. Similarly, although precision medicine has made significant progress in breast and other well-studied cancers, its application in PDAC remains limited. This highlights the need for further research into the use of nanoparticles for more effective and targeted treatment strategies. The rational design of polymer-based nanoparticles for PDAC therapy requires careful consideration of polymer type, nanoparticle architecture, and targeting strategies. Advances in polymer chemistry and nanotechnology are enabling the development of multifunctional nanoplatforms that can navigate the hostile PDAC microenvironment, deliver drugs selectively, and overcome biological barriers. Continued interdisciplinary research integrating materials science, cancer biology, and drug delivery will be essential to translating these systems from bench to bedside for the management of pancreatic cancer.

## Data Availability

The original contributions presented in the study are included in the article/supplementary material, further inquiries can be directed to the corresponding author.
